# Increased Femoral Anteversion Does Not Lead to Increased Joint Forces During Gait in a Cohort of Adolescent Patients

**DOI:** 10.3389/fbioe.2022.914990

**Published:** 2022-06-06

**Authors:** Nathalie Alexander, Reinald Brunner, Johannes Cip, Elke Viehweger, Enrico De Pieri

**Affiliations:** ^1^ Laboratory for Motion Analysis, Department of Paediatric Orthopaedics, Children’s Hospital of Eastern Switzerland, St. Gallen, Switzerland; ^2^ Department of Orthopaedics and Traumatology, Cantonal Hospital St. Gallen, St. Gallen, Switzerland; ^3^ Laboratory for Movement Analysis, University of Basel Children’s Hospital, Basel, Switzerland; ^4^ Department of Paediatric Orthopaedics, University of Basel Children’s Hospital, Basel, Switzerland; ^5^ Dpartment of Biomedical Engineering, University of Basel, Basel, Switzerland; ^6^ Department of Paediatric Orthopaedics, Children’s Hospital of Eastern Switzerland, St. Gallen, Switzerland

**Keywords:** femoral torsion, coxa antetorta, musculoskeletal modelling, joint loading, in-toeing, hip internal rotation, knee flexion

## Abstract

Orthopedic complications were previously reported for patients with increased femoral anteversion. A more comprehensive analysis of the influence of increased femoral anteversion on joint loading in these patients is required to better understand the pathology and its clinical management. Therefore, the aim was to investigate lower-limb kinematics, joint moments and forces during gait in adolescent patients with increased, isolated femoral anteversion compared to typically developing controls. Secondly, relationships between the joint loads experienced by the patients and different morphological and kinematic features were investigated. Patients with increased femoral anteversion (*n* = 42, 12.8 ± 1.9 years, femoral anteversion: 39.6 ± 6.9°) were compared to typically developing controls (*n* = 9, 12.0 ± 3.0 years, femoral anteversion: 18.7 ± 4.1°). Hip and knee joint kinematics and kinetics were calculated using subject-specific musculoskeletal models. Differences between patients and controls in the investigated outcome variables (joint kinematics, moments, and forces) were evaluated through statistical parametric mapping with Hotelling T2 and t-tests (α = 0.05). Canonical correlation analyses (CCAs) and regression analyses were used to evaluate within the patients’ cohort the effect of different morphological and kinematic predictors on the outcome variables. Predicted compressive proximo-distal loads in both hip and knee joints were significantly reduced in patients compared to controls. A gait pattern characterized by increased knee flexion during terminal stance (KneeFlex_*tSt*_) was significantly correlated with hip and knee forces, as well as with the resultant force exerted by the quadriceps on the patella. On the other hand, hip internal rotation and in-toeing, did not affect the loads in the joints. Based on the finding of the CCAs and linear regression analyses, patients were further divided into two subgroups based KneeFlex_*tSt*_. Patients with excessive KneeFlex_*tSt*_ presented a significantly higher femoral anteversion than those with normal KneeFlex_*tSt*_. Patients with excessive KneeFlex_*tSt*_ presented significantly larger quadriceps forces on the patella and a larger posteriorly-oriented shear force at the knee, compared to patients with normal KneeFlex_*tSt*_, but both patients’ subgroups presented only limited differences in terms of joint loading compared to controls. This study showed that an altered femoral morphology does not necessarily lead to an increased risk of joint overloading, but instead patient-specific kinematics should be considered.

## Introduction

Femoral anteversion refers to the twist between the proximal and distal parts of the femur on the transverse plane (Kaiser et al., 2016). The normal amount of torsion depends on age and sex (Hefti, 2000; Jacquemier et al., 2008), starting approximatively at 40° of anteversion at birth, and decreasing to 15–20° during adulthood (Crane, 1959; Fabry et al., 1973). When increased femoral anteversion does not resolve spontaneously during growth and persists during adolescence, it can be associated with disturbances in mobility, such as an in-toeing gait pattern, and represents a frequent reason for consultation with pediatric orthopedic clinicians (Fabry, 2010). There is no consistent definition in the literature of what is considered as pathologically increased femoral anteversion, with values ranging from >30° to 50° (Jani et al., 1979; Cordier and Katthagen, 2000; Hefti, 2000).

Although increased femoral anteversion is often considered a primarily cosmetic problem, it can also be associated with orthopedic and functional problems in pediatric and adolescent patients, who may eventually require surgical intervention. Increased femoral torsion was reported to lead to functional problems, especially concerning high falling frequencies (Leblebici et al., 2019; Mackay et al., 2021) and altered lower-limb kinematics during gait, such as in-toeing and increased hip internal rotation (Bruderer-Hofstetter et al., 2015; Passmore et al., 2018; Alexander et al., 2019; Mackay et al., 2021). Since femoral anteversion measured by computed tomography (CT) scans correlates weakly with hip internal rotation during walking, a complete three-dimensional (3D) gait analysis was recommended when planning a surgical correction of torsional deformities (Radler et al., 2010; Gaston et al., 2011). However, gait deviations due to increased femoral anteversion in otherwise typically developing adolescents often receive little attention as increased femoral anteversion <50° is most of the time not considered for surgery (Tonnis and Heinecke, 1999; Cordier and Katthagen, 2000; Sass and Hassan, 2003; Fabry, 2010). These gait deviations may nevertheless cause future complaints, such as limited function and pain (Mackay et al., 2021). Corrective osteotomies for increased femoral anteversion are usually conducted in patients during adolescence. While femoral derotation osteotomy is a common procedure in patients with cerebral palsy (Dreher et al., 2007; Dreher et al., 2012), the indication is less common in otherwise typically developing children and adolescents (MacWilliams et al., 2016).

Nevertheless, previous studies have also shown an association between femoral anteversion and anterior knee pain (Eckhoff et al., 1997), as the rotational alignment of the limb has a major impact on patellar kinematics (Keshmiri et al., 2016). Powers (2003) and Stevens et al. (2014) reported a correlation of increased femoral anteversion with patellofemoral pain, while increased femoral anteversion is also a known risk factor for patellofemoral instability (Dejour and Le Coultre, 2007). Lower limb torsional malalignment is also one of the major risk factors implicated in the development of overuse injuries (Pagliazzi et al., 2022). Femoral torsional and coronal deformities have previously been correlated with hip pain and labral damage (Tönnis and Heinecke, 1999). A recent meta-analysis of 1756 patients found a positive correlation between increased anteversion and the severity of hip osteoarthritis (Parker et al., 2021). Excessive femoral anteversion was also associated with femoroacetabular impingement (FAI) syndrome (Ito et al., 2001; Audenaert et al., 2012; Ejnisman et al., 2013). Ejnisman et al. (2013) investigated hips with FAI and reported that patients with femoral anteversion greater than 15° were 2.2 times more likely to have labral tears. Furthermore, alterations of the femoral torsional morphology could affect the orientation of the hip intra-articular forces (De Pieri et al., 2021), which could be associated with the spatial distribution of the acetabular cartilage damage observed during adulthood, especially in concomitance with other morphological alterations, such as cam and pincer deformities that characterize FAI syndrome (Pascual-Garrido et al., 2019).

This evidence suggests the need for a more comprehensive understanding of the influence of increased femoral anteversion on hip and knee joint loading. Since surgical corrections are taken into consideration for pediatric and adolescent patients before the onset of secondary orthopedic problems, it is particularly important to investigate potentially pathological joint mechanics in this specific demographic cohort. In order to gain a better understanding of hip and knee joint loading, musculoskeletal modelling can be used to assess intra-articular loads while accounting for both subject-specific morphological and kinematic characteristics (De Pieri et al., 2021). Passmore et al. (2018) compared hip and knee loading using generic and subject-specific musculoskeletal models in a cohort of adolescent patients with increased femoral anteversion and increased tibial torsion, and predicted increased mediolateral patellofemoral joint contact forces when using subject-specific models. In a heterogeneous cohort of 37 healthy and asymptomatic adults (range: −7° retroversion to +38° anteversion), De Pieri et al. (2021) found significant correlations between higher femoral anteversion and higher anterior (swing phase) and medial (loaded stance phase) hip contact forces during gait. Furthermore, modelling patient-specific femoral anteversion was also shown to be particularly important for the analysis of patients affected by cerebral palsy, who often present torsional bony deformities (Kainz et al., 2020; Kainz et al., 2021; Veerkamp et al., 2021). These studies highlight the importance of accounting for subject-specific morphological information in the analysis of joint loads. However, a direct comparison of joint loading between patients with increased femoral anteversion and healthy controls is still lacking in literature, thus not allowing drawing any clinically relevant conclusion regarding the effect of these morphological deviations on joint mechanics and the long-term risk of joint overloading.

In addition to an altered bone morphology, patients with increased femoral anteversion also tend to present altered joint kinematics during gait. Previous studies have shown that these patients tend to walk with a more internally-rotated foot progression angle, increased hip internal rotation, increased hip flexion and greater anterior pelvic tilt. Additionally, an increased knee flexion in mid- and terminal stance was found for some children with increased femoral anteversion (Bruderer-Hofstetter et al., 2015; Passmore et al., 2018; Alexander et al., 2019; Mackay et al., 2021). In general, children with different lower-limb torsional deformities, tend to present kinematic compensatory mechanisms (Alexander et al., 2020; Byrnes et al., 2020). A stratified analysis based on different kinematic gait patterns might help identifying specific subgroups of patients who present a higher risk of joint overloading as a consequence of both altered morphology and kinematics.

It was hypothesized that pediatric and adolescent patients with increased femoral anteversion present increased hip and knee joint contact forces during gait compared to controls, which could be associated with some of the orthopedic complications previously reported for these patients. Furthermore, different loading situations, based on the specific deviations of their gait pattern, were expected. Therefore, the first aim of this study was to investigate lower-limb kinematics and kinetics, as well as intra-articular joint forces during gait in pediatric and adolescent patients with increased femoral anteversion compared to typically developing controls. Secondly, relationships between the joint loads experienced by the patients and different morphological and kinematic features were investigated. Specifically, the effects of femoral anteversion, the midpoint of hip rotational range of motion, and different kinematic characteristics representative of the gait patterns were analyzed through regression analyses. Finally, when potential indicators of joint overload were identified, a further subgroup analysis based on the specific gait pattern was carried out.

## Methods

### Participants

**FIGURE 1:**
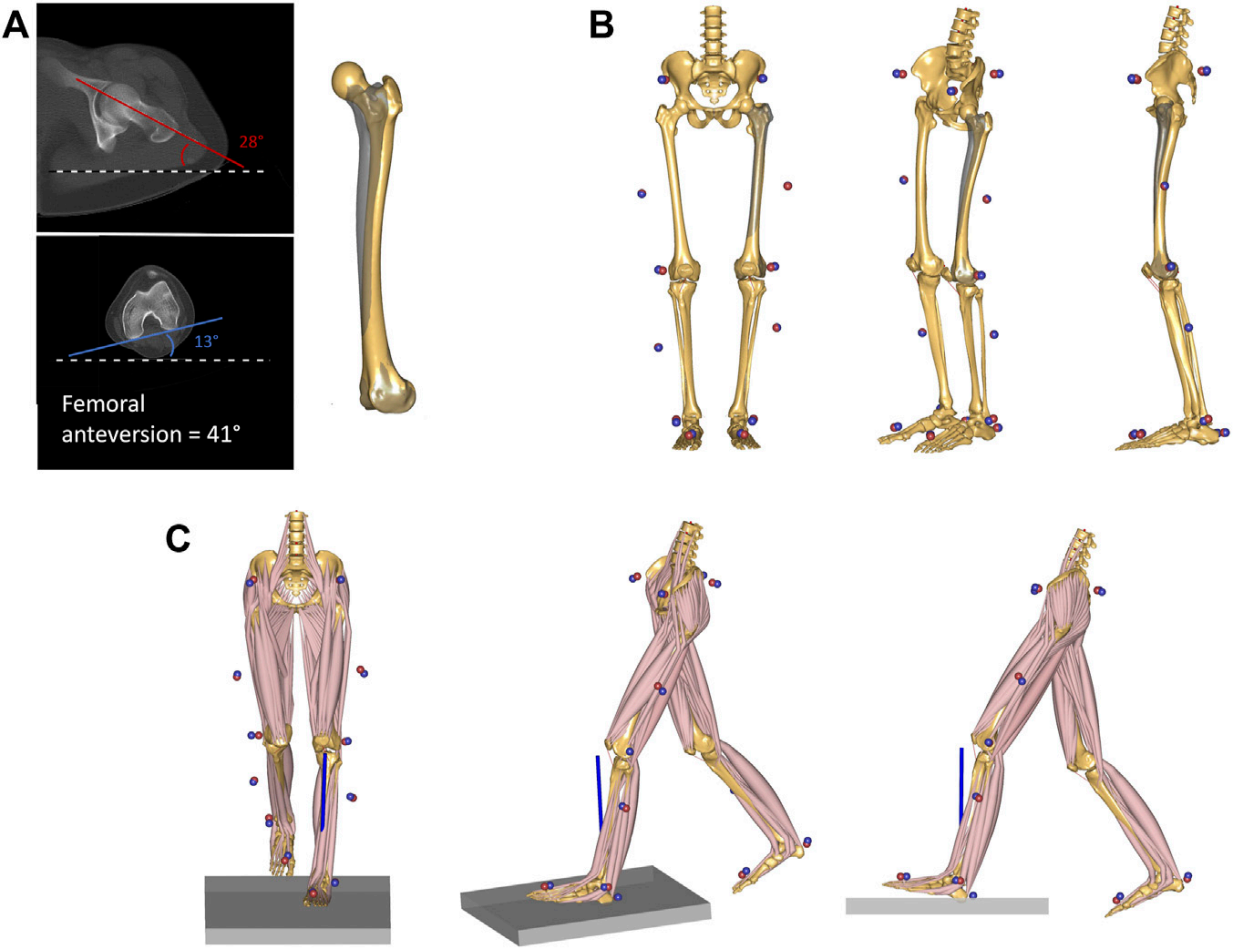
**(A)** CT-measurement of femoral anteversion in one of the investigated patients. The angle between the horizontal reference (black and white dashed line) and both femoral neck axis (red) and the posterior contour of the femoral condyles (blue) were measured. The femoral anteversion angle is reported as a sum of the two values. The femoral geometry of the musculoskeletal model was then personalized to match the measured torsional value. The original unscaled femoral geometry is shown in shaded grey, while the personalized geometry is shown in solid yellow. **(B)** The model was scaled to match the subject’s anthropometrics based on marker data collected during a standing reference trial. The measured markers’ data are presented as blue spheres, while the virtual markers attached to the musculoskeletal model are shown as red spheres. **(C)** Kinematic and kinetic analyses during gait were based on the tracking of the measured marker trajectories and the ground reaction forces. An inverse dynamics analysis based on a third-order-polynomial muscle recruitment criterion was performed to calculate required muscle activations, as well as resulting joint moments and contact forces.

Forty-two patients with increased femoral anteversion were retrospectively included from the overall patient pool at the Children’s Hospital of Eastern Switzerland (Table 1). Patients with CT-confirmed femoral anteversion >30° were included in the study. Femoral anteversion was calculated as the angle between the femoral neck axis and the posterior contour of the femoral condyles (Figure 1) (Hernandez et al., 1981) from CT measurements (Somatom Definition AS 64 slices, Siemens Healthineers, Erlangen, Germany). Patients’ data were compared to control data of nine healthy children and adolescents collected at the University of Basel Children’s Hospital (Table 1), for whom normal values of femoral anteversion were confirmed through existing MRI measurements (Siemens Prisma, Siemens Healthineers, Erlangen, Germany) to limit radiation exposure. Exclusion criteria were defined as follows for patients and controls: <8 years or >18 years, leg length discrepancy >1 cm, any kind of foot deformity, tibiofemoral varus/valgus deformity >5°, adiposity (body mass index over 90th percentile), scoliosis or any type of psychomotor or neurological disorder, pathological tibial torsion; additional for controls: pathological femoral anteversion. The study was approved by the regional ethics board (Ethics Committee Northwest Switzerland EKNZ 2021-00015) and written informed consent was provided by all participants and their legal guardians.

**TABLE 1:** Mean (standard deviation) anthropometric data and walking speed for patients and controls, as well as distinct gait variables for patients including (range).

	Patients (n = 42)	Controls (n = 9)	*p*-value
Femoral anteversion [°]	39.6 (6.9) [30–63]	18.7 (4.1) [13–25]	0.000
Midpoint _*HipRot ROM*_ [°]	24.6 (10.5) [0–40]	—	—
Age [years]	12.8 (1.9) [10.3–17.5]	12.0 (3.0) [8.5–16.3]	0.270
Gender [female/male]	26/16	5/4	—
Height [m]	1.56 (0.10) [1.40–1.78]	1.53 (0.18) [1.28–1.77]	0.520
Mass [kg]	44.9 (9.5) [30.8–68.6]	41.8 (12.3) [28.3–63.2]	0.413
Walking speed [m/s]	1.25 (0.11) [0.93–1.50]	1.32 (0.23) [1.06–1.61]	0.142
**Specific gait pattern descriptors**			
HipRot_*tSt*_ [°]	14.0 (6.9) [3.4–29.1]	—	—
KneeFlex_*tSt*_ [°]	15.4 (4.5) [3.3–24.2]	—	—
FootProg_*tSt*_ [°]	1.9 (5.8) [−11.2–12.7]	—	—

Midpoint _*HipRot ROM*_, Midpoint of passive hip rotation range of motion (positive value indicate internal rotation); HipRot_*tSt*_, mean hip rotation in terminal stance; KneeFlex_*tSt*_, mean knee flexion in terminal stance; FootProg_*tSt*_, mean foot progression angle in terminal stance.

### Data Collection

Self-reflecting markers were attached according to the PiG-model (Kadaba et al., 1990). Participants walked barefoot at a self-selected normal speed. Lower-limb kinematics and kinetics were collected during gait using a 3-dimensional motion capture system (Vicon Motion Systems Ltd., Oxford, United Kingdom, 200 Hz), and force plates (patients: AMTI, Advanced Mechanical Technology Inc., Watertown, Massachusetts, United States, 1,000 Hz; controls: Kistler Instrumente AG, Winterthur, Switzerland, 1,000 Hz). Proper marker placement was checked using a static and dynamic trial prior to the measurement with a primary focus on the correct placement of the knee and thigh markers. Knee ab-/adduction motion >15° during swing phase indicated imprecise marker placement (Reinschmidt et al., 1997). A minimum of three valid gait cycles were collected, where participants hit the force plates without any visual interruption of the gait cycle.

Clinical examination was further conducted on patients and included amongst others evaluation of hip and knee passive range of motion. Clinical hip rotation is presented as mid-point of hip rotational range of motion (Midpoint _*HipRot ROM*_), which is mid-point between maximal internal and external hip rotation (Kerr et al., 2003). If the Midpoint _*HipRot ROM*_ is positive, hip internal rotation predominates while for negative values hip external rotation predominates.

### Musculoskeletal Modelling

Data were first processed using Vicon Nexus (Vicon, Oxford Metrics Ltd., Oxford, United Kingdom). Following, marker trajectories and ground reaction forces (GRF) were filtered using a second-order low-pass Butterworth filter with a cut-off frequency of 5 and 12 Hz, respectively, and used as input for an inverse dynamics analysis in the AnyBody Modelling System (version 7.3, AnyBody Technology A/S, Aalborg, Denmark) (Damsgaard et al., 2006). Personalized models for each subject were created from a detailed generic model of the lower limb (De Pieri et al., 2018), based on a cadaveric dataset (Carbone et al., 2015), scaled to match the overall anthropometrics and the marker data collected during the static standing reference trial (Lund et al., 2015). The geometry of the femur was morphed to include a transversal rotation between the proximal and distal sections, matching the subject’s femoral torsion value obtained from the imaging data (De Pieri et al., 2021) (Figure 1). The hip joints were modelled as 3-degrees of freedom (DoF) ball-and-socket joints, while knee and talocrural joints were modelled as 1-DoF hinges. The position of the patella was defined as a function of the knee flexion angle, and motion of the subtalar joint was restricted due to the reduced number of markers on the foot segment (one heel and one toe marker). The muscle elements were modelled with a simple muscle model represented by constant strength actuators.

Joint kinematics were computed from the measured marker trajectories and reported according to the International Society for Biomechanics’ (ISB) recommendations (Wu et al., 2002). The foot progression angle relative to the direction of gait was also calculated. The orientation of the foot was identified through an axis connecting the heel and the second-metatarsal markers, while the direction of gait was defined as the line connecting the positions of the heel marker in two consecutive ipsilateral heel strikes.

An inverse dynamics analysis based on a third-order-polynomial muscle recruitment criterion was then performed to calculate required muscle activations and forces, as well as resulting joint moments and contact forces (Andersen, 2021). Hip moments and hip contact forces (HCFs) were calculated in a proximal (pelvis-based) coordinate system according to ISB recommendations (Derrick et al., 2020). Knee moments and knee contact forces (KCFs) were computed in an anatomical tibia-based coordinate system similar to (Grood and Suntay, 1983) based on the bony landmarks of the tibial plateau. The resultant force exerted by the quadriceps muscles on the patella was also computed (De Pieri et al., 2022).

### Data Analysis

Gait trials were processed and analyzed through the toolkit AnyPyTools (Lund et al., 2019) in the Python programming language (Python Software Foundation, Wilmington, DE, United States). The analysis was limited to the affected leg for the patients (or the leg with the highest measured femoral anteversion in case of bilateral involvement), while a randomly chosen leg was analyzed for the control group. Joint moments were normalized to body mass, while joint contact forces were normalized to body weight (BW). Average trajectories per subject were then calculated based on the collected walking trials. Kinematic trajectories (angles) were time-normalized to the gait cycle (GC) from foot-strike (0%) to foot-strike (100%) of the leg of interest, and kinetic trajectories (moments and forces) were time-normalized to the stance phase (ST), from foot-strike (0%) to foot-off (100%) of the leg of interest.

### Comparison Between Patients and Controls

Differences between patients and controls in anthropometrics, femoral anteversion, clinical examination values, and walking speed were assessed using independent Student’s *t*-tests with a significance level set at α = 0.05. Moreover, the time profiles of joint kinematics, moments and contact forces were analyzed using statistical parametric mapping (SPM; www.spm1D.org, v0.43) (Pataky, 2012). The three hip joint angles were regarded as a 3D vector field, describing the 3D spatial variation of the kinematic vector trajectory over time. The use of vector field analysis takes into consideration covariance between spatial components, thus reducing errors due to covariation bias (Pataky et al., 2013). Similarly, hip moments, hip contact forces, and knee contact forces were described as 3D vectorial fields. Knee flexion angle, foot progression angle, knee sagittal moment, and the resultant force of the quadriceps acting on the patella were considered as separate 1D time-dependent scalar variables. Comparisons between patients and controls were carried out as SPM-based two-sample Hotelling T2 tests for 3D vector fields and as two-sample, two-tailed t-tests for 1D scalar variables. The output test statistics, SPM(T2) or SPM(t), were evaluated at each point of the gait cycle or stance phase. The significance level was set a priori at α = 0.05, and the corresponding critical thresholds T2* and t* were calculated based on the temporal smoothness of the input data through random field theory. In case of 3D vector field analysis, post-hoc scalar t-tests were also conducted using on each separate component, with Bonferroni-corrected significance threshold levels set at α = 0.05/3 = 0.017.

### Regression Analyses With Morphological and Kinematic Parameters

For the second aim, canonical correlation analyses (CCAs) and regression analyses were used to evaluate within the patients’ cohort the effect of different morphological and kinematic predictors on the investigated outcome variables, meaning joint kinematics, moments, and forces during gait. The SPM-based analyses allowed to identify specific intervals of the gait cycle or stance phase in which the outcome variables were correlated with the individual predictors. Femoral anteversion and the clinically assessed Midpoint _*HipRot ROM*_ were used as morphological independent variables for the analyses. Furthermore, three distinct kinematic characteristics were chosen to identify potential relationships between subject-specific gait patterns and the outcome variables. The distinct kinematic gait characteristics were hip internal rotation, knee flexion, and foot progression angle. Synthetic values, defined for each variable as the mean value during terminal stance (31–50% GC), were used as independent variables in the regression analyses: HipRot_*tSt*_, KneeFlex_*tSt*_ and FootProg_*tSt*_, respectively (Table 1). Finally, walking speed was also included as an independent variable. For each independent variable, SPM-based independent CCAs or scalar linear regressions were used to identify potential correlations with 3D vector fields and 1D scalar outcome variables, respectively. The significance level was set at α = 0.05. When a statistically significant correlation was observed between one of the independent variables and a 3D vector fields, post-hoc scalar linear regressions were also conducted on each separate component, with Bonferroni-corrected significance threshold levels set at α = 0.05/3 = 0.017.

### Subgroup Analysis

In order to further evaluate the clinical meaningfulness of these correlations, the patients were further divided into subgroups based on gait-pattern-specific characteristics whenever a significant correlation between a specific kinematic feature and the joint contact forces was detected. Patients were divided into two subgroups (e.g. based on KneeFlex_*tSt*_): one group characterized by values that fell within the normal range of the control data, and one group by values that excessively deviated from this range. The normative range was defined by identifying the mean value during terminal stance across controls. Each patient’s mean value during terminal stance was defined as exceeding control data when above/below the control’s mean ±1 standard deviation. Differences in anthropometrics, femoral anteversion, clinical examination values, gait speed, and distinct gait characteristics in terminal stance between subgroups were identified using independent Student’s *t*-tests. Furthermore, potential differences in joint kinematics, moments, and forces between each subgroup of patients (normal and excessive) and controls, as well as in-between subgroups, were analyzed through SPM-based two-sample Hotelling T2 tests for 3D vector fields and as two-sample, two-tailed t-tests for 1D scalar variables (α = 0.05, with post-hoc Bonferroni-corrected t-tests α = 0.017).

In the interest of clarity, for all SPM analyses only statistically significant differences in intervals longer than 2% of the gait cycle or stance phase are discussed.

## Results

### Comparison Between Patients and Controls

Patients presented a significantly larger femoral anteversion compared controls (+20.9°), while there were no statistically significant differences in terms of age, mass, height, and walking speed (Table 1).

**FIGURE 2:**
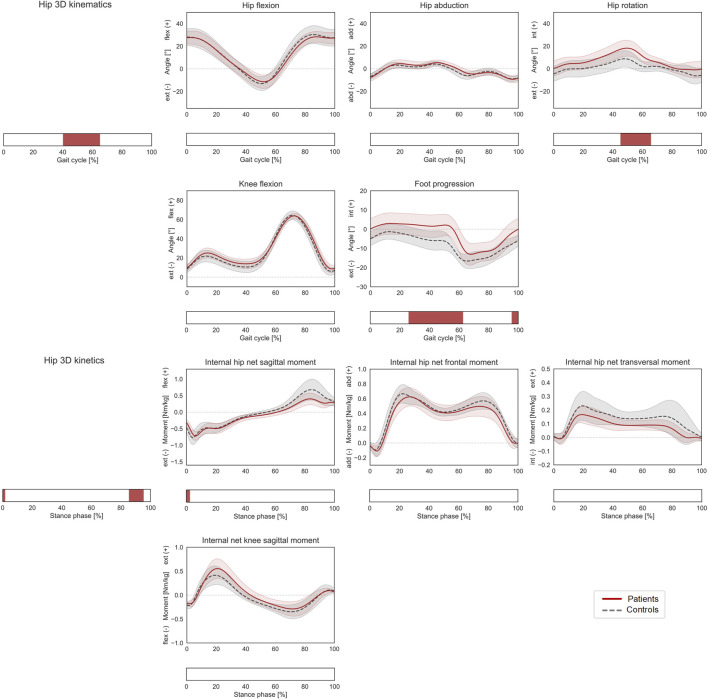
Lower-limb kinematics during the gait cycle (GC) and joint moments during stance phase (ST). Mean ± 1SD hip flexion, hip abduction, hip rotation, knee flexion, and foot progression angles, as well as hip sagittal, frontal, transversal, and knee sagittal moments, are reported as red solid lines for patients, and as grey dashed lines for controls. Intervals of GC or ST with a statistically significant difference in the relevant SPM tests are reported as red bars. Hip joint angles and moments were regarded as a 3D vector fields. 3-dimensional significant differences between patients and controls were analyzed by means of two-sample Hotelling T2 tests and are reported in the leftmost panels. 1D scalar outcome variables, as well as individual components of the vector fields in the post-hoc vectorial analyses, were analyzed by means of two-sample t-tests and intervals characterized by significant differences between the two groups are reported below each subplot.

**FIGURE 3:**
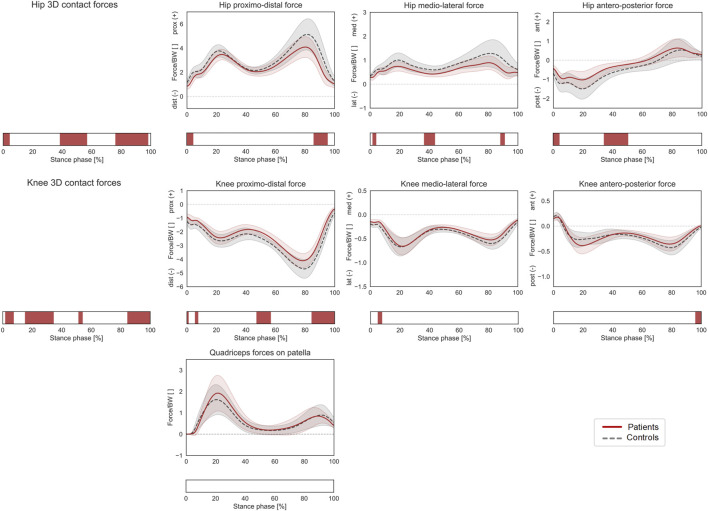
Joint forces during stance phase (ST). Mean ± 1SD hip and knee proximo-distal, medio-lateral, and antero-posterior contact forces, as well as resultant force exerted by the quadriceps muscles on the patella, are reported as red solid lines for patients, and as grey dashed lines for controls. Intervals of ST with a statistically significant difference in the relevant SPM tests are reported as red bars. Knee and hip contact forces were regarded as a 3D vector fields. 3-dimensional significant differences between patients and controls were analyzed by means of two-sample Hotelling T2 tests and are reported in the leftmost panels. The resultant force on the patella, as well as individual components of the vector fields in the post-hoc vectorial analyses, were analyzed by means of two-sample t-tests and intervals characterized by significant differences between the two groups are reported below each subplot.

Patients with increased femoral anteversion walked with significantly different hip 3D kinematics from terminal stance until initial swing (40–65% GC), which was mostly associated with an increased internal hip rotation in patients (post-hoc: 45–66% GC). The gait of the patients was also characterized by a more internally rotated foot progression angle from mid-stance to toe-off as well as in terminal swing (26–63% and 96–100% GC, Figure 2). The overall 3D hip joint moments showed significant differences briefly during heel-strike (0–2% ST) as well as in pre swing (86–95% ST), but post-hoc analyses only revealed a significantly lower hip extensor moment at initial contact for patients (0–2% ST). In terms of HCFs, statistically significant 3D differences were observed between the two groups during loading response, mid and terminal stance and pre swing (0–4%, 39–57%, and 76–98% ST). The post-hoc analysis revealed that patients presented lower proximal (0–4% and 86–95% ST), lower medial (2–4%, 37–44%, and 88–91% ST), and lower posterior (0–4% and 34–50% ST) hip joint forces compared to controls. Significant differences were also found in terms of KCFs (2–7%, 15–35%, 52–54%, and 85–100% ST). When looking at the individual force components, patients presented reduced distal (compressive) forces (6–8%, 47–57% and 84–100% ST), but only limited differences in terms of medio-lateral (5–8% ST) and antero-posterior (96–100%) shear components (Figure 3).

### Regression Analyses With Morphological and Kinematic Parameters

**FIGURE 4:**
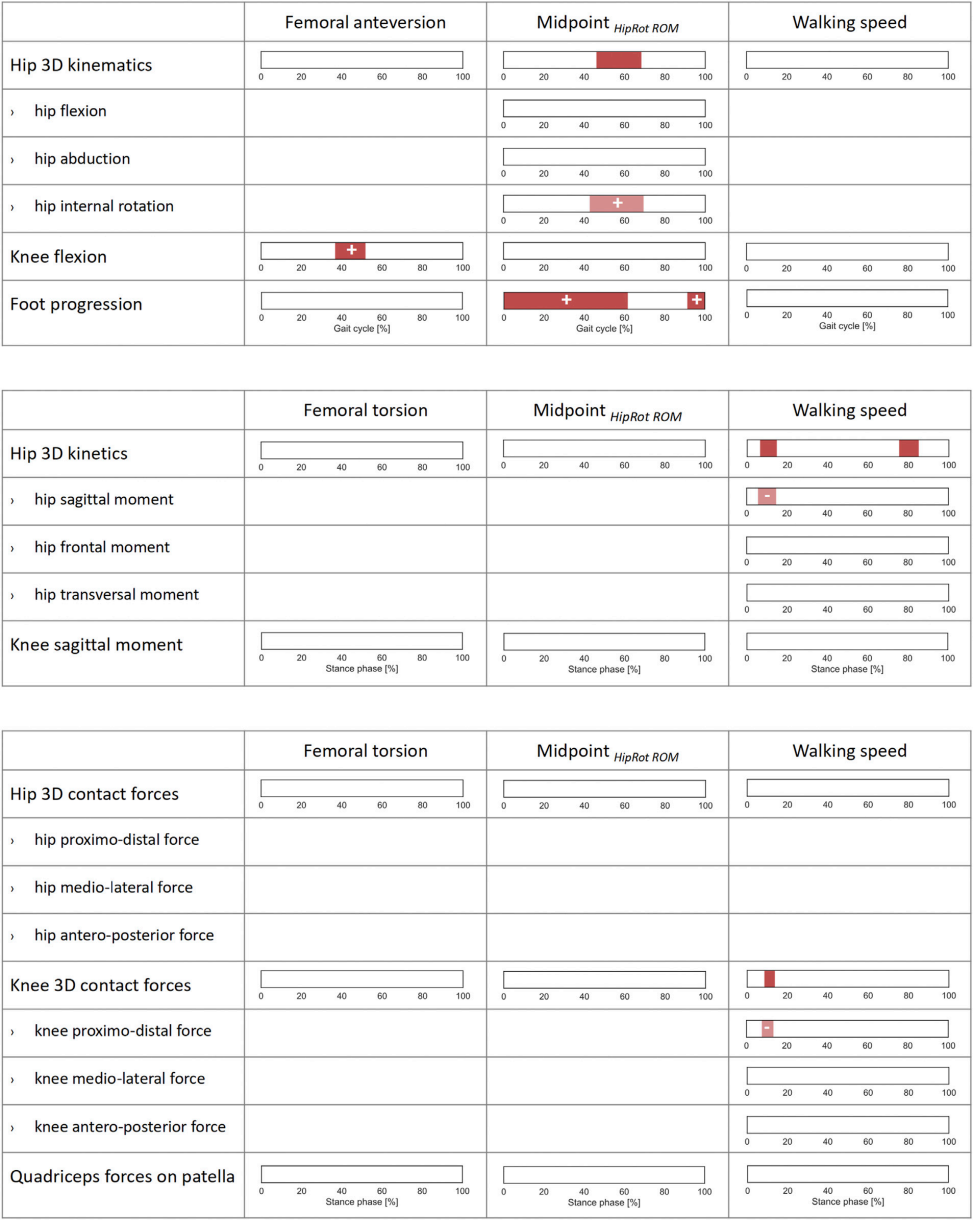
Correlations of lower-limb kinematics, joint moments, and joint forces with femoral anteversion, midpoint of hip rotational range of motion (Midpoint _*HipRot ROM*_), and walking speed across the patients’ cohort. SPM canonical correlation analyses (CCAs) or scalar linear regressions were used to identify potential correlations of the 3D vector fields and 1D scalar outcome variables (rows) with the independent predictors (columns). Intervals of the gait cycle for kinematics and intervals of the stance phase for moments and forces which are characterized by a statistically significant correlation in the relevant SPM test are reported as red bars. Post-hoc scalar linear regressions were conducted on the individual components of the vector fields, for which intervals with significant correlations are reported in light red. The sign of the correlation is indicated on each interval for all scalar linear regressions. For a correct interpretation of the directionality of the correlation, the reader is referred to the axes reported in the corresponding graphs in Figures 1, 2.

Within the patients’ cohort, femoral anteversion was only positively correlated with the knee flexion angle during terminal stance (37–52% GC). Midpoint _*HipRot ROM*_ was significantly correlated with the 3D hip joint angles from terminal stance to initial swing (46–68% GC). The post hoc linear regression analyses indicated a positive correlation between the Midpoint _*HipRot ROM*_ and hip internal rotation (43–69% GC). The foot progression angle also presented a positive correlation from terminal swing throughout stance phase (0–62%, and 91–100% GC). Walking speed correlated with the 3D hip moments during loading response and parts of terminal stance/pre-swing (7–15%, and 75–85% ST). The post-hoc analysis revealed that higher walking speeds were correlated with more extensive moments in the sagittal plane (6–14% ST). No correlation was found with the resulting joint forces (Figure 4).

**FIGURE 5:**
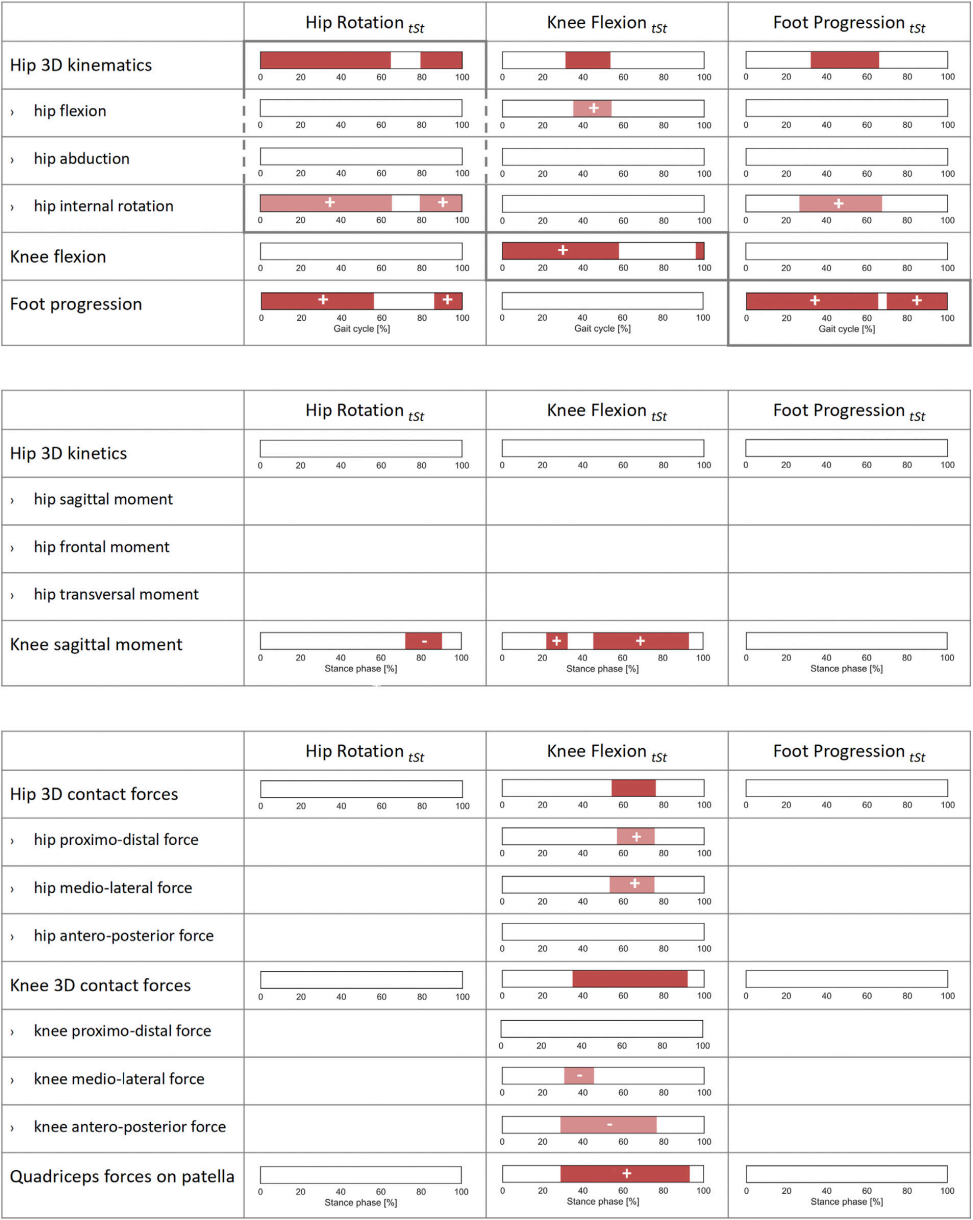
Correlations of joint kinematics, joint moments, and forces with synthetic gait-pattern-related kinematic descriptors (HipRot_*tSt*_, KneeFlex_*tSt*_, FootProg_*tSt*_, defined, respectively as the mean value of hip rotation, knee flexion, and foot progression during terminal stance) across the patients’ cohort. SPM canonical correlation analyses (CCAs) or scalar linear regressions were used to identify potential correlations of the 3D vector fields and 1D scalar outcome variables with the independent predictors. Intervals of the gait cycle for joint kinematics, and intervals of the stance phase for joint moments and forces, characterized by a statistically significant correlation in the relevant SPM test are reported as red bars. Post-hoc scalar linear regressions were conducted on the individual components of the vector fields, for which intervals with significant correlations are reported in light red. The sign of the correlation is indicated on each interval for all scalar linear regressions. For a correct interpretation of the directionality of the correlation, the reader is referred to the axes reported in the corresponding graphs in Figures 1, 2.

HipRot_*tSt*_ was correlated with the overall hip 3D kinematics from mid-swing throughout stance phase (0–64% and 79–100% GC). The post hoc linear regression analyses indicated a positive correlation with the hip internal rotation angle itself (0–65% and 79–100% GC). Additionally, HipRot_*tSt*_ was correlated with the foot progression angle from mid-swing until terminal stance phase (0–56% and 86–100% GC). It was also observed that a more internally rotated hip is correlated with a higher sagittal knee flexion moment during late stance (72–90% ST), however no correlation with joint forces was found (Figure 5).

KneeFlex_*tSt*_ correlated well with the knee flexion angle itself throughout stance phase and in terminal swing (0–58% and 96–100% GC). KneeFlex_*tSt*_ was correlated with the overall hip 3D kinematics in terminal stance (31–53% GC) with the post hoc linear regression analyses indicating a positive correlation with hip flexion (35–54% GC). In terms of joint kinetics, KneeFlex_*tSt*_ was positively correlated with the sagittal knee moment (22–33% and 45–93% ST) as well as overall 3D KCFs (35–92% ST) and 3D HCFs (54–76%). The post hoc linear regression analyses indicated that higher values of KneeFlex_*tSt*_ were associated with larger proximal (compressive) (57–75% ST) and medial (53–75% ST) HCFs, and with more laterally (31–45% ST) and posteriorly oriented (29–76% ST) KCFs. Additionally, KneeFlex_*tSt*_ was correlated with the quadriceps force on the patella (29–93% ST) (Figure 5).

FootProg_*tSt*_ correlated with the foot progression angle itself throughout most of the gait cycle (0–65% and 70–100% GC), and it also presented a positive correlation with hip internal rotations from terminal stance to initial swing (26–68% GC). No correlation with joint moments or forces were found (Figure 5).

### Subgroup Analysis

**FIGURE 6:**
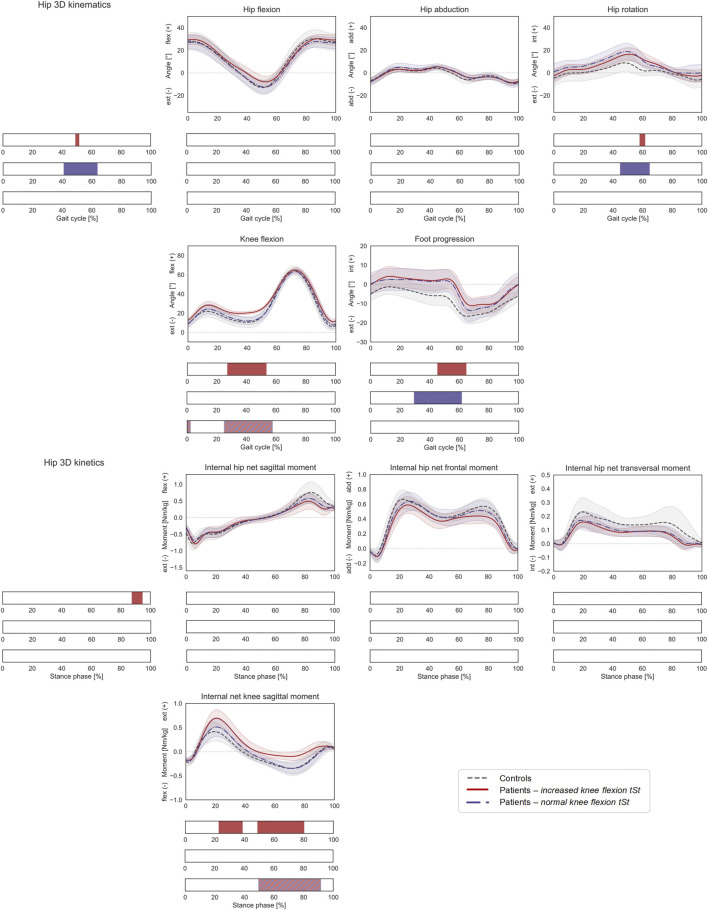
Lower-limb kinematics during the gait cycle (GC) and joint moments during stance phase (ST) with patient stratification according to knee flexion angle during terminal stance (KneeFlex_*tSt*_). Mean ± 1SD hip flexion, hip abduction, hip rotation, knee flexion, and foot progression angles, as well as hip sagittal, frontal, transversal, and knee sagittal moments, are reported as grey dashed lines for controls, as red solid lines for patients with increased KneeFlex_*tSt*_, and as blue dashed-dotted lines for patients with normal KneeFlex_*tSt*_. Intervals of GC or ST with a statistically significant difference in the relevant SPM tests are reported as red bars for the comparison between controls and patients with increased KneeFlex_*tSt*_, as blue bars for the comparison between controls and patients with normal KneeFlex_*tSt*_, and as red-and-blue striped bars for the comparison between patients with increased vs. normal KneeFlex_*tSt*_. Hip joint angles and moments were regarded as a 3D vector fields. 3-dimensional significant differences between patients and controls were analyzed by means of two-sample Hotelling T2 tests and are reported in the leftmost panels. 1D scalar outcome variables, as well as individual components of the vector fields in the post-hoc vectorial analyses, were analyzed by means of two-sample t-tests and intervals characterized by significant differences between the two groups are reported below each subplot.

Based on the finding of the CCAs and linear regression analyses, patients were further divided into two subgroups based on KneeFlex_*tSt*_. Patients with excessive KneeFlex_*tSt*_ had also significantly higher femoral anteversion than those with normal KneeFlex_*tSt*_ (Table 2). The gait of patients with excessive KneeFlex_*tSt*_ was characterized by a larger knee flexion at heel contact and from mid stance to pre-swing (0–2% and 25–58% GC), as well as by a more extensive knee sagittal moment from mid stance to pre swing (49–92% ST) (Figure 6). In terms of 3D KCFs, significant differences were found between the two groups during terminal stance (52–85% ST). The post-hoc analysis indicated that patients with excessive KneeFlex_*tSt*_ present significantly larger posterior-oriented shear forces (50–78% ST), in addition to a large overall force exerted by the quadriceps on the patella (46–92% ST).

**TABLE 2:** Anthropometrics and clinical examination for sub-grouping scenario based on knee flexion in terminal stance. Values are presented as means (standard deviation).

Grouping scenario	KneeFlex_*tSt*_		
	Normal (*n* = 32)	Excessive (*n* = 10)	*p*-value
Femoral anteversion (°)	37.6 (5.7)	44.9 (7.8)	0.030
Height (m)	1.56 (0.11)	1.55 (0.07)	0.452
Mass (kg)	45.3 (10.4)	43.7 (6.3)	0.393
Age (years)	12.8 (1.9)	13.0 (1.9)	0.968
Walking speed (m/s)	1.25 (0.12)	1.25 (0.11)	0.347
**Clinical examination**			
Hip external rotation (°)	17.2 (12.4)	11.5 (12.0)	0.088
Hip internal rotation (°)	63.4 (14.8)	70 (15.1)	0.773
Midpoint _*HipRot ROM*_ (°)	23.1 (9.9)	29.3 (11.5)	0.279
Hip extension (°)	9.4 (4.0)	9.0 (6.6)	0.921
Hip flexion (°)	136.1 (9.1)	134 (10.2)	0.193
Knee extension (°)	4.5 (4.3)	4.0 (3.2)	0.773
Knee flexion (°)	157.0 (5.8)	158.5 (3.4)	0.538
Popliteal angle (°)	30.6 (16.2)	21.0 (15.6)	0.935

Midpoint _*HipRot ROM*_, Midpoint of passive hip rotation range of motion (positive value indicate internal rotation); KneeFlex_*tSt*_, mean knee flexion in terminal stance.

**FIGURE 7:**
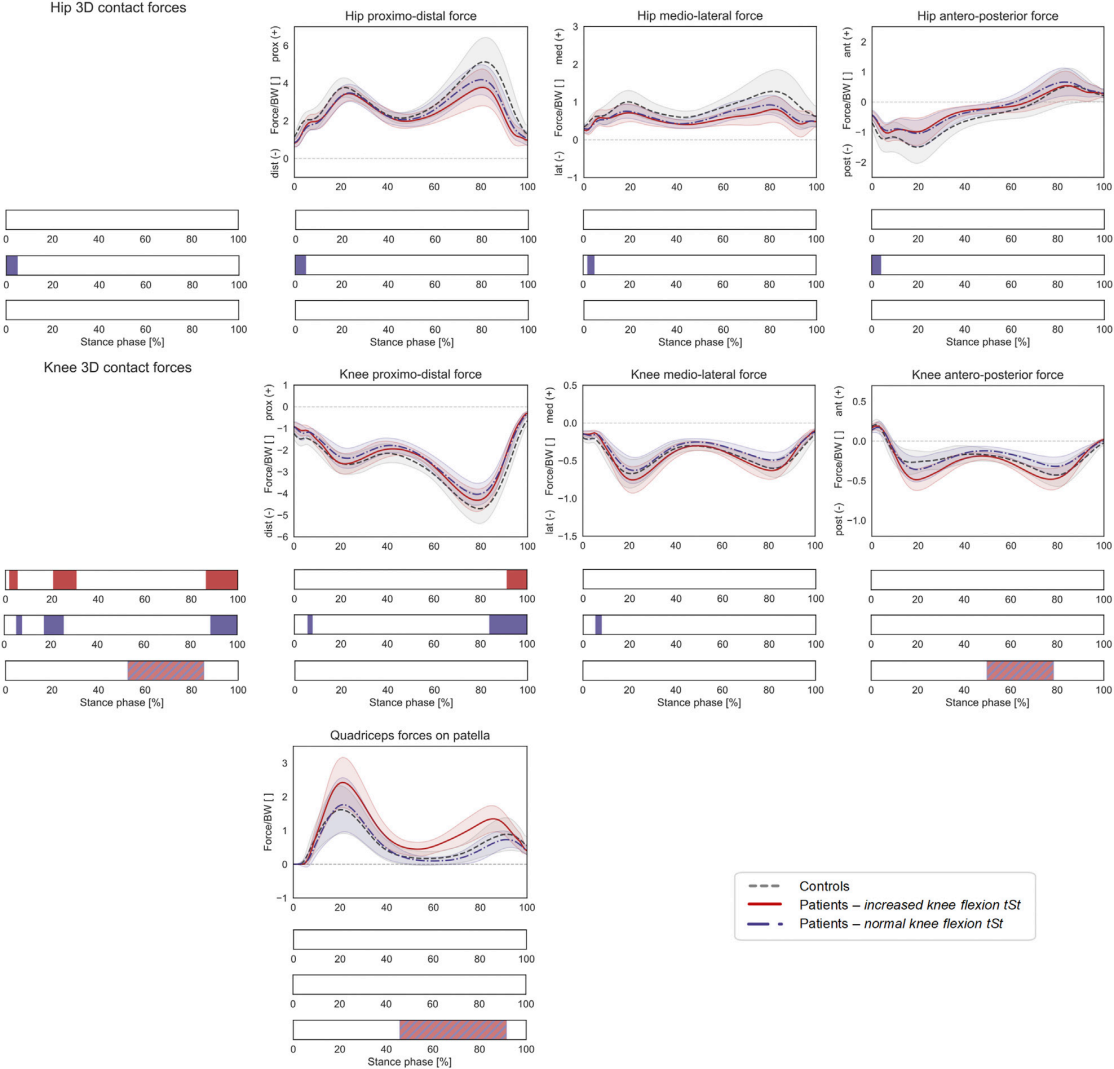
Joint forces during stance phase (ST) with patient stratification according to knee flexion angle during terminal stance (KneeFlex_*tSt*_). Mean ± 1SD hip and knee proximo-distal, medio-lateral, and antero-posterior contact forces, as well as resultant force exerted by the quadriceps muscles on the patella, are reported as grey dashed lines for controls, as red solid lines for patients with increased KneeFlex_*tSt*_, and as blue dashed-dotted lines for patients with normal KneeFlex_*tSt*_. Intervals of ST with a statistically significant difference in the relevant SPM tests are reported as red bars for the comparison between controls and patients with increased KneeFlex_*tSt*_, as blue bars for the comparison between controls and patients with normal KneeFlex_*tSt*_, and as red-and-blue striped bars for the comparison between patients with increased vs. normal KneeFlex_*tSt*_. Knee and hip contact forces were regarded as a 3D vector fields. 3-dimensional significant differences between patients and controls were analyzed by means of two-sample Hotelling T2 tests and are reported in the leftmost panels. The resultant force on the patella, as well as individual components of the vector fields in the post-hoc vectorial analyses, were analyzed by means of two-sample t-tests and intervals characterized by significant differences between the two groups are reported below each subplot.

Compared to healthy controls, patients with excessive KneeFlex_*tSt*_ walked with a more flexed knee (27–53% GC), a more internally rotated hip (post-hoc: 58–62% GC), a more internally rotated foot progression angle (45–65% GC), and a larger internal knee net extensive moment (23–39% and 49–80% ST). No statistically significant differences were found in terms of HCFs (Figure 7), while the 3D KCFs differed significantly between the two groups (2–5%, 20–30% and 86–100% ST). However, the post-hoc analysis only revealed significant differences during pre-swing (91–100% ST) in the proximo-distal component. Despite presenting larger quadriceps forces on the patella, no statistically significant differences compared to controls were found.

On the other hand, patients with a normal KneeFlex_*tSt*_ walked with more a pronounced hip internal rotation compared to controls (45–65% GC), as well as with an increased foot progression angle (29–62% GC). Statistically significant differences in HCFs were observed during heel strike for the 3D vector field (0–5% ST) as well as for the individual components (proximo-distal: 0–5% ST, medio-lateral 0–5% ST, antero-posterior 0–4%). KCFs also significantly differed in terms of the overall vector field (5–8%, 20–30% and 86–100% ST), the proximo-distal component (5–8% and 84–100% ST), and the medio-lateral component (5–8% ST).

## Discussion

This study investigated lower limb joint loads during gait in pediatric and adolescent patients with increased, isolated femoral anteversion. This was done by: 1) comparing patients to typically developing controls; 2) analyzing within the patients’ cohort potential correlations between morphological parameters, kinematic gait descriptors, and joint loads; 3) stratifying the patients according to specific features of their gait pattern.

### Comparison Between Patients and Controls

The hypothesis that children with increased femoral anteversion present increased joint loads during gait had to be rejected, as the predicted compressive proximo-distal loads in both hip and knee joints were significantly reduced in patients compared to controls. Significant differences in the 3D HCF and KCF vectors were limited to narrow intervals of the loaded stance phase. Furthermore, the analysis of the individual force components showed limited differences and overall similar trends, suggesting also a similar intra-articular orientation of the joint contact forces in both hip and knee (Figure 2).

The overall gait pattern of the investigated cohort of patients, characterized by increased internal hip rotation and in-toeing, is in agreement with previous research focusing on the gait of adolescent patients with increased femoral anteversion (Bruderer-Hofstetter et al., 2015; Passmore et al., 2018; Alexander et al., 2019; Mackay et al., 2021).

Increased internal hip rotation during walking has been previously discussed as a compensatory mechanism to restore the abducting capacity of the hip abductors, which present a reduced abducting lever arm for femoral morphologies characterized by a large anteversion (Arnold et al., 1997; De Pieri et al., 2021). Although the patients included in this study presented an excessive femoral anteversion (range: 30–63°), their gait was not characterized by significant differences in the internal hip net frontal moment compared to controls (Figure 1). This suggests that they were able to produce a comparable abduction moment around the hip during gait, and that their overall hip-abductive capacity is not compromised during this activity.

### Regression Analyses With Morphological and Kinematic Parameters

Within the patients’ cohort, different morphological and kinematic predictors of potential joint over-loading were explored through independent CCAs and regression analyses. A gait pattern characterized by increased knee flexion during terminal stance was found to be significantly correlated with the intra-articular forces acting on hip and knee, as well as with the resultant force exerted by the quadriceps on the patella. In particular, increased KneeFlex_*tSt*_ is associated with increased medial and proximal (compressive) HCFs, increased lateral and posterior (shear) KCFs, as well as increased quadriceps force on the patella during the loaded stance phase (Figure 4). On the other hand, the commonly reported transversal plane gait deviations, HipRot_*tSt*_ and FootProg_*tSt*_, did not affect the loads in the joints. No significant correlations between femoral anteversion and joint loads were found, in contrast to a previous study reporting higher medial HCFs for larger femoral torsional values during stance phase in a cohort of healthy asymptomatic adults (De Pieri et al., 2021).

In terms of kinematics, no significant correlation between femoral anteversion and hip rotation during walking was found. This is in agreement with previous studies showing a weak correlation between femoral anteversion and hip rotation, as a result of a considerable dynamic influence of compensatory mechanisms during walking (Radler et al., 2010; Gaston et al., 2011; Braatz et al., 2013; Mackay et al., 2021; Schranz et al., 2021). Additionally, in case of flat feet, the oblique axis of rotation in the hind foot could lead to hip internal rotation when the foot is loaded (Zafiropoulos et al., 2009), which would have to be further considered for a comprehensive assessment of overall lower-limb alignment. However, in this study only patients without foot deformities were included. However, a significant correlation between femoral anteversion and knee flexion in terminal stance was observed (Figure 3).

Midpoint _*HipRot ROM*_ was significantly correlated with hip internal rotation and the foot progression angle. In agreement with Kerr et al. (2003), Midpoint _*HipRot ROM*_ is a better indicator for transversal gait deviations than femoral anteversion. Even though lower-limb kinematics were affected by the Midpoint _*HipRot ROM*_, there was no statistically significant effect on joint loading and the clinical rotational ability did not influence the hip-abductor moment either.

Walking speed was also included as an independent variable in regression analyses, as it is known to influence joint kinematics, kinetics and intra-articular forces during gait (Schwartz et al., 2008; De Pieri et al., 2019; Schreiber and Moissenet, 2019). However, hardly any correlations between kinematics and kinetics were found. Therefore, discussed differences can be accounted on morphological or kinematic gait differences rather than walking speed differences.

The synthetic gait pattern descriptors HipRot_*tSt*_, KneeFlex_*tSt*_ and FootProg_*tSt*_, defined as mean values during terminal stance, showed extensive correlations with the corresponding kinematic parameters from which they were derived. HipRot_*tSt*_ and FootProg_*tSt*_ were positively correlated with hip rotation and foot progression angles, respectively, through most of the gait cycle, indicating that these are good synthetic indicators for an internally rotated hip and inward foot progression angle during gait. In contrast, KneeFlex_*tSt*_ is a good indicator for the knee flexion in the loaded stance phase, but not during swing. Furthermore, HipRot_*tSt*_ was correlated with the foot progression angle, and conversely FootProg_*tSt*_ was correlated with the hip internal rotation angle, suggesting that these two variables are coupled and representative of a transversal plane kinematic mechanism. A larger KneeFlex_tSt_ on the other hand was also correlated with a larger hip flexion during terminal stance, suggesting that adaptations at multiple joint levels in the sagittal plane can occur. Two different kinematic mechanisms, one in the transversal and one in the sagittal plane, seem therefore to exist. However, the knee sagittal moment during stance was correlated with both HipRot_*tSt*_ and KneeFlex_*tSt*_, suggesting that both transversal and sagittal kinematic deviations can affect sagittal plane functionality.

A reduced efficacy of the plantar flexor-knee extension couple mechanism (Passmore et al., 2018) was previously reported as possible cause for increased knee flexion while in-toeing. Within the investigated cohort however, we could not observe any significant correlation between the foot progression angle and knee flexion in terminal stance, suggesting that, in patients with solely increased femoral anteversion, in-toeing does not impair knee extension during gait. Interestingly, with increasing hip internal rotation the knee flexor moment in terminal stance increased even though with increasing femoral anteversion an increase in knee flexion during terminal stance was found (e.g. increasing KneeFlex_*tSt*_ lead to a decreased knee flexor moment). Therefore, hip internal rotation might be a compensatory mechanism to achieve an adequate knee flexor moment in terminal stance. This hypothesis, however, needs further evaluation and even though only patients without foot deformities were included in the current study, the effect of foot kinematics on knee flexion might has to be considered since as, e.g., increased forefoot supination can be linked to several gait parameters (Pothrat et al., 2013).

### Subgroup Analysis

When stratifying the patients in two groups according to their knee flexion during terminal stance (the only kinematic parameter showing significance in the regression analyses), patients with excessive KneeFlex_*tSt*_ presented significantly larger quadriceps forces on the patella and a larger posteriorly-oriented shear force at the knee, compared to patients with normal KneeFlex_*tSt*_ (Figure 6). Nevertheless, both patients’ subgroups presented only limited differences in terms of joint loads compared to controls suggesting only limited clinical relevance of different gait patterns in terms of joint loading. Increased quadriceps force on the patella is in line with reported increasing patellofemoral compression forces increasing knee flexion angle (Modenese et al., 2013; Alexander and Schwameder, 2016) and the fact that increased quadriceps force contributes to larger tibiofemoral and patellofemoral joint loadings with increasing knee flexion (Steele et al., 2012). Patients with increased KneeFlex_*tSt*_ showed significantly reduced knee flexor moments in terminal stance compared to controls as well as patients with normal KneeFlex_*tSt*_, in contrast to a previous study where the same group of patients was compared to a different control group (Alexander et al., 2019).

In this groups, increased knee flexion in terminal stance was not caused by a decreased knee joint range of motion since patients could achieve full knee extension with a bias towards knee hyper-extension (Table 2) as previously reported in the literature (Passmore et al., 2018; Alexander et al., 2019). Furthermore, besides higher femoral anteversion in patients with excessive KneeFlex_*tSt*_, no differences concerning anthropometrics or clinical examination values were found (Table 2).

### Limitations and Outlook

Finally, the following limitations should be considered. Patients with increased femoral anteversion were included based on clinical evaluation of hip rotation, which was verified in CT-scans, possibly introducing a selection bias. Only data for a limited number of patients and controls having femoral anteversion verified *via* CT or MRI scans was available. An analysis of a larger and more heterogeneous sample of patients is warranted to further confirm the validity of these findings and potentially identify critical subgroups of patients who might not have been represented in this study. For instance, increased femoral anteversion is a comorbidity commonly observed in patients with cerebral palsy, where the impaired muscular control combined with structural deformities could lead to more pronounced functional impairments (Kainz et al., 2021). Additionally, future studies should also include patients with foot deformities since correlations between flat feet and hip internal rotation have been previously reported (Zafiropoulos et al., 2009) and foot deformities might lead to further gait deviations as well.

Due to the limited number of patients and controls, individual independent regression analyses rather than a multiple regression were performed, thus not allowing assessing any interaction between predictors, which were instead treated as independent variables. Nevertheless, all chosen morphological and gait-pattern-related predictors have a direct applicability in clinical practice, making this analysis a relevant starting point for identifying potential factors associated with a risk of joint overloading in patients with increased femoral anteversion.

Personalized musculoskeletal models accounting for radiographically-measured femoral torsional values were created for all subjects. However, anatomical variations in the neck-shaft angle could also affect the resulting HCFs (Kainz et al., 2020). HCFs were calculated in a proximal (pelvis-based) coordinate system according to ISB recommendations. While this standardization enables comparisons with other studies, calculating HCFs in an anatomically-oriented reference frame according to subject-specific acetabular inclination and version could further reveal intra-patient differences and potentially help identifying critical loading scenarios at the hip. A correct understanding of the orientation of the HCFs in the acetabulum could be even more relevant for activities other than gait, which present substantially different HCFs (Lunn et al., 2020).

Furthermore, simplified ankle and knee joint mechanics, characterized by one DoF in each joint, were implemented in the models, neglecting subtalar version, knee transversal rotation, as well as knee varus or valgus alignment. Bretin et al. (2011) on the other hand showed in a cadaver study that hip internal rotation resulted in valgus deviation of the mechanical axis of the lower limb, and in a shift of the center of force towards the lateral condyle of the knee. Furthermore, quadriceps forces were assessed as an indicator of the loads sustained by the patella, which could however be underestimated for patients who present altered patellar contact mechanics. Future studies should therefore include a more complex multi-DoF model of the femorotibial and femoropatellar joints, including a detailed characterization of the articular contact geometry and the stabilizing passive soft-tissue constraints (Lenhart et al., 2015; Marra et al., 2015; Dejtiar et al., 2020). A more detailed understanding of knee and patellar mechanics could be particularly important in the analysis of patients with miserable malalignment syndrome, in which an increased femoral anteversion and a concomitant outward tibial torsion can cause anterior knee pain, patellar maltracking and instability (Eckhoff, 1994; Eckhoff et al., 1997; Bruce and Stevens, 2004; Stevens et al., 2014; Pagliazzi et al., 2022).

Finally, muscles fascicles were modelled as constant strength actuators, neglecting force-length and force-velocity relationships. Using a Hill-type muscle model would require accurate knowledge of muscle-tendon properties, which were not available for this group of participants, and could have therefore introduced a bias in the models’ predictions (Arnold et al., 2013; Carbone et al., 2016). Future studies should aim at devising experimental protocols to calibrate these properties over subject-specific ranges of motion (Heinen et al., 2016) and consider muscle weakness in the presence of altered femoral morphology (Vandekerckhove et al., 2021).

Based on the current results increased femoral anteversion by itself might not be a problem concerning joint overloading. The effect of femoral torsional deformities should be assessed in a holistic biomechanical analysis of the patient, which accounts for patient-specific kinematics, clinical examination of joint mobility, as well as potentially coexisting bony deformities, such as cam and pincer morphologies, increased tibial torsion, or altered patellar morphology. A static analysis of altered femoral morphology alone is not sufficient for a correct management of the patient, but should rather be framed in a more dynamic assessment that takes into account motion and muscle functionality. Within this context, a patient-specific assessment of joint loads through gait analysis and musculoskeletal modelling represents an important tool for a well-informed management of orthopedic conditions associated with altered bony morphologies. Future studies based on larger datasets and machine learning algorithms could help identifying clinical, anatomical, and movement-related parameters for a rapid and data-driven estimation of joint loading, thus assisting orthopedic clinicians in assessing the patient-specific risks of further orthopedic complications (Burton II et al., 2021; Mouloodi et al., 2021; Xiang et al., 2021).

## Conclusion

Torsional deformities of the lower limb have been associated with a number of orthopedic complications, leading to the overall assumption in current clinical practice that an altered morphology is problematic. Through the analysis of a cohort of pediatric and adolescent patients with isolated increased femoral anteversion, this study showed that an altered femoral morphology does not always lead to an increased risk of joint overloading. When taking into account both subject-specific morphological and kinematic deviations, the investigated patients presented hip and knee loads within normative values, or even slightly reduced. Increased femoral anteversion by itself might therefore not always be a problem warranting orthopedic intervention. Nevertheless, the presence of other orthopedic problems, such as additional morphological deformities, and their potential interplay with increased femoral anteversion should still be kept in mind in the clinical management of these patients.

## Data Availability

The datasets presented in this article are not readily available because of privacy restrictions. Requests to access the datasets should be directed to enrico.depieri@unibas.ch.
